# Effects of a healthy food supply intervention in a military setting: positive changes in cereal, fat and sugar containing foods

**DOI:** 10.1186/1479-5868-9-91

**Published:** 2012-07-31

**Authors:** Clarissa ML Bingham, Marjaana Lahti-Koski, Pauli Puukka, Marja Kinnunen, Piia Jallinoja, Pilvikki Absetz

**Affiliations:** 1 Health Behavior and Health Promotion Unit, Department of Lifestyle and Participation, National Institute for Health and Welfare, P.O. Box 30, FI-00271, Helsinki, Finland; 2 Finnish Heart Association, Oltermannintie 8, P.O. Box 50, 00621, Helsinki, Finland; 3 Department of Chronic Disease Prevention, National Institute for Health and Welfare, Peltolantie 3, FI-20720, Turku, Finland; 4 National Consumer Research Center, P.O. Box 5, (Kaikukatu 3), 00531, Helsinki, Finland

**Keywords:** Food habits, Food intake, Intervention, Health promotion, Military, Soldiers

## Abstract

**Background:**

In Finland, all men are liable to military service and a clear majority completes service. The increasing prevalence of obesity also among soldiers concerns conscripts’ food choices. Conscripts are served nutritionally planned regular main meals but individual choices take place in free-time eating. This study assesses the effects in conscripts’ eating habits in an intervention targeting the supply of healthy foods available in the military setting.

**Methods:**

Participants were 604 18-21-year old male conscripts of whom 242 belonged to Control Group and 362 to Intervention Group. Participants of Control Group were historical controls performing military service one year before Intervention Group. The intervention targeted selection, placement, and attractiveness of healthy foods in garrison refectories and soldier’s home cafeterias, the two main food providers in the military. Dietary intake data was collected by self-administered questionnaire at three time points: before/beginning of military service (T0), 8 weeks (T1) and 6 months (T2) of military service. Outcome measures were food consumption frequencies and four dietary indexes (Cereal Index, Fruit and Vegetable Index, Fat Index and Sugar Index) developed to characterize the diet. Changes between study groups in outcome variables and in time were analysed by repeated-measures analysis of covariance.

**Results:**

Significant (p < 0.05) intervention effects and time-intervention interactions mostly in favor of Intervention Group were found. In Intervention Group, Cereal Index was significantly higher at T2 and the overall level of porridges and cereals was higher during follow-up when comparing to Control Group. Also, the overall levels of Fat Index, potato chips, soft drinks and desserts as well as sweet pastries at T1 were significantly lower in Intervention Group. At the same time, Fruit and Vegetable Index and the level of fruit and berries were lower in Intervention Group during follow-up.

**Conclusions:**

In the military setting, healthier food choices can be promoted by intervening on the main food environments by improving the supply of healthy foods. However, impacting on conscripts’ individual selection as fruit and vegetable consumption is more challenging.

## Background

In the military environment, soldiers need to meet fitness and body composition standards to meet health and physical requirements of military service [1]. Still, overweight and obesity are increasing in armed forces as in civilian populations [2]. Among Finnish conscripts, average body weight has increased in 15 years from 71 to 77 kg while height has remained constant [3,4]. The majority of conscripts is normal weight but one third has a BMI of over 25 kg/m^2^[5-7]. A similar pattern is evident elsewhere too: in the United States, even more than half of men are overweight for enlistment [8]. The proportion of men in active duty with a BMI of over 30 kg/m^2^ has almost tripled in 1995–2008 [9]. In the UK, 14% of soldiers have a BMI of over 30 kg/m^2^[10].

Together with anthropometrics, diet should also be considered. In general, Finnish conscripts’ intakes of energy-yielding nutrients are close to recommendations [7,11]. Still before military service, only 13% of young men consumed daily vegetables and as few as 8% fruits and berries [12]. In service, choices for energy-rich, nutrient-poor foods, such as pizza and soft drinks, prevail [11]. Consumptions of sweet foods, i.e. desserts, doughnuts and confectionary, increase compared to pre-service levels [13-15]. Positively however, the consumption of rye bread increases and that of some fatty foods such as French fries remains stable or even decreases [13].

In Finland, compulsory military service applies to all men aged 18–29 years and service is usually entered at 19–20 years. The duration is 6, 9 or 12 months and arrivals enter in January and in July. Nearly 80% of each age cohort complete service [16]. The rest either apply for non-military service or are exempted because of medical reasons [17].

Military service is a period of institutional life where actions are externally directed and schedules ordered. Possibilities of making independent decisions are limited also in eating. Complete freedom of dietary choices is unfeasible although some individual selection is possible in free-time. Soldiers are offered regular nutritionally-planned main meals [11] in a structured environment which offers a good setting for intervening. To our knowledge there have been only a few interventions targeting eating habits and obesity in military populations [2]. A central topic in the existing interventions has been supporting lifestyle or behavioral change also through a supportive environment [18-22]. Specifically intervening in soldiers’ nutrition through dietary education has been focal [18-20,22-26]. Furthermore, nutrition labelling [27] and increased serving of fruit and vegetables at a garrison refectory [28] have been experimented as intervention strategies.

Although Finland is a country with compulsory military service and a long tradition of successful community based interventions targeting healthy eating [29-32], the controlled military environment has been dismissed. We conducted an intervention targeting the supply of healthy foods available to conscripts. The aim of this study is to assess the effects of the intervention in conscripts’ eating habits with consumptions of fiber, fat and sugar containing foods as well as four food indexes as outcome measures.

## Methods

### Design

This study was conducted as a part of the DefenceNutri study which is a controlled two-phase intervention trial aiming at improving conscripts’ eating habits [33]. The study took place during three years 2007–9. Control group data was collected in 2007. This data served also as a needs’ assessment data for the intervention taking place in 2008 and 2009. We present results of Control Group and the first intervention year (Intervention Group). Our design has two arms with 3 measurement time points each.

### Setting

The study took place in two garrisons: Armoured Brigade and Kainuu Brigade. Armoured Brigade is situated in Southern Finland, and conscripts serving there live in cities, towns and rural areas in Southern and central Finland. Kainuu Brigade in North-Eastern Finland recruits men from Western, central and Northern Finland and they live in cities, semi-urban or rural areas. The study protocol was approved by the ethics committee of the Hospital District of Helsinki and Uusimaa.

Military training takes place in garrison and encampment conditions. At garrison, daily service lasts approximately 10 hours. At encampment, service is intensive and may take place around the clock. Eating is enabled and food provided by the military catering organization which also operates dining halls i.e. refectories at garrisons. Daily breakfast, lunch and dinner form a part of compulsory service. Also a voluntary evening snack is available on most days. This food is planned and prepared according to particular military nutrition recommendations for conscripts. It is calculated to cover nutritional requirements of service and offers a varied diet [11]. The menu of the refectories is fixed: lunch and dinner contain one main dish served with fresh or cooked vegetables. Bread, especially rye crisp bread, is always available. Desserts, such as fruit soup and pudding, are served daily at meals. Drink alternatives include milk, sour milk, juice, and water. At meals in the refectory, conscripts may choose which components to eat and their quantity but not the contents of food served. During free-time outside service, conscripts can buy subsidized affordable snacks, such as sweets, soft drinks and sandwiches, inside the garrison from soldier’s home cafeterias operated by paid personnel and voluntary workers. To summarize, military eating takes for the most part place at the garrison refectory and in free-time at the soldier’s home cafeteria. Together these two form the main eating environments of military service. Additionally in free-time, conscripts’ can exit the garrison and purchase food from surrounding grocery shops and restaurants or order delivered food.

### Intervention

The intervention objective was to increase the supply of healthy foods at the two main eating environments in the military setting, garrison refectories and soldier’s homes cafeterias. The specific nutritional goals were: to increase fruit and vegetable consumption; to increase fiber intake; to decrease the intake of fat and especially saturated fat; and to decrease sugar intake.

In autumn 2007, guided workshops for the core personnel of garrison refectories and soldier’s homes cafeterias were carried out separately at both garrisons. These were conducted with the aim of developing action plans for promoting healthy food supply and to reach the nutritional goals of the intervention. The 4–6 participants from each organization, respectively, were experts of their operational environment and thus well suitable for development work. The participants of the garrison refectory included supervisors, cooks and members of catering staff, while participants of the soldier’s homes cafeterias were the manager, paid personnel and voluntary workers.

**Figure 1:**
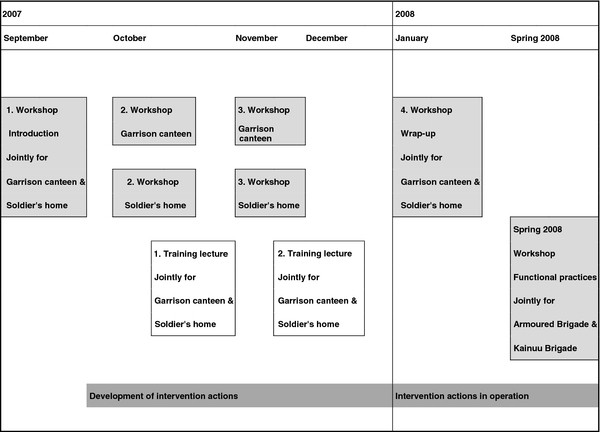
Progression of the healthy food supply intervention.

The first workshop was arranged jointly for the garrison refectory and soldier’s home cafeteria personnel for orientation and co-operation between the two organizations. The second and third workshops were organized separately for the two organizations in order to maximize feasibility of the developed action plans in their respective operational environments. A fourth workshop was organized jointly for both personnel for evaluation of action plans, implementation of successful plans, and establishing further developmental work. Additionally in spring 2008, a final workshop jointly for both study garrisons was conducted to share ideas and compare action plans. Two sets of training lectures were organized in connection with workshops to support the development work and to increase personnel’s motivation in health promotion. The first training lectures focused on promoting conscripts’ health and covered the meaning of healthy eating in the military setting, increasing attractiveness of healthy food, and profitability calculations. The second lectures focused on promoting health of the personnel of garrison refectories and soldier’s homes cafeterias. The progression of the intervention is illustrated in Figure 1.

In the course of the workshops, experiments of improving the food supply were conducted at the garrison refectories and soldier’s home cafeterias to test which changes and improvements are feasible. Full-scale implementation of intervention actions took place as the supply intervention in 2008. Intervention actions contained apparent actions which can be measured quantitatively as well as less-visible actions to improve the quality of the food supply. Examples of both types of realized intervention actions are listed in Table 1. Outside of the focus of this study in 2009, the intervention targeted conscripts, with the objective of increasing the demand of healthy food choices and especially vegetable consumption.

**Table 1:** Examples of actions of the healthy food supply intervention phase

**Apparent quantitatively measurable actions**	**Less-visible actions to improve quality of food supply**
**Garrison refectories**	**Garrison refectories**
Serving fruit (apples and oranges) cut into slices instead of whole ones	Eliminating all usage of butter except in one traditional meat dish
Stopping serving sugar-sweetened juice at all meals	Replacing white pasta and rice with dark fiber-rich varieties
Increasing serving selection of fiber-rich bread such as rye bread	Adding bran to porridges
**Soldier's home cafeterias**	**Soldier's home cafeterias**
Replacing normal and big bags of sweets by small ones and dried fruit near cashier	Converting white bread dough of own bakery into more fiber-rich by adding e.g. bran, seeds
Including fresh vegetables in all sandwich, bun etc. fillings	Replacing sandwich fillings from fatty meat e.g. sausage to lean meat cuts
Developing small sweet pastries to be sold	Replacing full-fat yoghurt by fat-free alternatives

### Participants

Control Group data was collected among men entering military service in three service units (i.e. companies, each containing from 80 to 150 men) in the study garrisons in January and July of 2007. Participants filled in questionnaires at three time points. The first questionnaire was sent to the men’s home address one month prior to service (T0). They responded by Internet or by returning the questionnaire when entering service. The second questionnaire was filled in at the end of the basic training period at 8 weeks of service (T1). The last follow-up questionnaire was filled in at six months of service (T2).

Intervention Group data was collected from men entering military service in two service units in January and July of 2008. For them, the study protocol was modified slightly due to changes in available study resources. Participants filled in the first questionnaire during the first week of military service but were instructed to answer questions on eating habits retrospectively according to civilian life (T0). The second (T1) and third (T2) questionnaires were filled in at the same time points as in Control Group.

In all, this study comprises the follow-up data of 604 men who filled properly in study questionnaires at all time points. The number of participants at different time points and their characteristics are presented in Table 2. The disparity between the proportion of Control Group (40.1%) and Intervention Group (59.9%) is explained by two factors: Firstly in Control Group, the first questionnaire at T0, which was sent home, could not be targeted specifically to men performing military service in the study units because placing to units is done at the beginning of military service. Secondly for Intervention Group, collaborating military personnel was more familiar with the study protocol and thus more motivated during the second year. Drop-out between the time points was due to: interruption of service, being on encampment, on leave or ill during measurements, military transfers to other units or garrisons, and refusals to attend the study. The last-mentioned applies particularly to the first questionnaire (T0) of Control Group which was sent home. Only participants aged less than 22 years were included to have a sample with a homogenous age range. Therefore 11 men in Control Group and 13 men in Intervention Group aged 22 or over were excluded from analyses. All participants gave their written informed consent.

**Table 2:** Characteristics of study participants (n = 604)

		**All**		**Control group**		**Intervention group**	
		**n**	**%**	**n**	**%**	**n**	**%**
Study group		604	100	242	40.1	362	59.9
Participation rate	Number of men in units at T0*	2688	100	1430	53.2	1258	46.8
	Respondents at T0	1502	55.9‡	645	42.9	857	57.1
	Respondents at T1	1634	60.8‡	926	56.7	708	43.3
	Respondents at T2	947	35.2‡	589	62.2	358	37.8
	Respondents at T0 + T1 + T2†	604	22.5‡	242	40.1	362	59.9
Month of military arrival	January	358	59.3	129	53.3	229	63.3
	July	246	40.7	113	46.7	133	36.7
Garrison	Armoured Brigade	313	51.8	84	34.7	229	63.3
	Kainuu Brigade	291	48.2	158	65.3	133	36.7
Age	18	1	0.2	1	0.4		
	19	131	21.8	65	27.0	66	18.4
	20	452	75.3	170	70.5	282	78.6
	21	16	2.7	5	2.1	11	3.1
Marital status	married or co-habiting	52	8.7	20	8.4	32	8.9
	single	545	91.1	217	91.6	328	90.9
	Widowed	1	0.2			1	0.3
Living status	alone	56	9.3	24	10.0	32	8.9
	with spouse	42	7.0	16	6.7	26	7.2
	with parents	485	81.0	198	82.5	287	79.9
	other	16	2.7	2	0.8	14	3.9

*T0 = beginning of military service.

† 11 men in Control Group and 13 men in Intervention Group being aged 22 or over were excluded to have a homogenous age range.

‡Percentage calculated from number of men in units at T0.

### Measures

Data on eating habits was collected with a 36-item food frequency questionnaire (FFQ) in which consumption was reported as number of days during the previous week [13,14]. The FFQ was applied from several corresponding validated questionnaires used in Finnish population studies [34-37]. Items in it were chosen to represent all major food groups of the Finnish diet. Background information on the menus of garrison refectories and from a detailed food diary study among Finnish conscripts [11] was used to design the FFQ to be best suited to the conscript study population. Thus, the purpose of the questionnaire was to identify overall quality of diet and food choices of young men performing military service. The study questionnaires included also other eating habit questions such as food choices as well as sociodemographic background, health behavior and psychosocial factors. Similarly as the FFQ, these questions were adopted from validated Finnish population study questionnaires [34,35,37].

The main outcome measures of this study are four food indexes, Cereal Index, Fruit & Vegetable Index, Fat Index and Sugar Index, which were formed to characterize important dimensions of the diet of these young men. Cereal Index was designed to represent cereal fiber intake as it was the sum of three healthy fiber-rich food items: weekly consumptions of rye bread, mixed bread, and porridges and cereals. Fruit & Vegetable Index was the sum of two items: fresh vegetables, and fruit and berries. Fat Index was the sum of five less-healthy fatty food items: meat pies and pastries, pizza and kebab, hot dogs and hamburgers, French fries, and potato chips. Sugar Index was the sum of five sugar-rich food items: desserts, sugared soft drinks, sweet pastries, chocolate and sweets. Food index items were given one point for each day the food was used during the previous week. The indexes were scaled by dividing the sum scores by the number of food items in each index. Thus, the total score of all indexes ranges between 0–7 and the results are comparable. Of the food items in the indexes, desserts are served at the garrison refectory where the other items are not typically provided but may be bought form soldier’s homes cafeterias and from grocery shops outside garrisons [11].

### Statistical analyses

First to review the data, descriptive statistics, including means, minimum, maximum values and standard deviations, were analyzed. Also, distributions were explored and clear outlier values were removed from the data. For outcome variables (food items and the four food indexes), differences in mean values between study groups were tested parametrically with independent samples t-tests at the three study time points (T0, T1, T2). Then within study groups, differences in mean values between time points (T0-T1, T0-T2) were tested parametrically with paired-samples t-tests.

To analyse the effect of the intervention, repeated-measures analysis of covariance (ancova) was conducted for each outcome variable separately. Food consumption frequency and food index values at each time point (T0, T1, T2) were inserted as dependent variables as within subject factors and the number of levels was set at 3. Study group (Control Group, Intervention Group) was entered as the between-subject factor and garrison as covariate. Also, adjustment for baseline value of each dependant variable was systemically performed [38]. Significant main effects for time and intervention as well as time-intervention interactions were explored and are reported. Also profile plots with estimated marginal means were drawn and these are presented for outcome variables with a significant time-intervention interaction. The critical p-value for significance was set at 0.05. All statistical analyses were conducted using PASW software version 17.

## Results

### Food consumption at baseline

Food consumption differed between Intervention Group and Control Group at baseline (T0). Fiber Index and Fruit and Vegetable Index as well as all food items in them except one were higher in Intervention Group than in Control Group (p ≤ 0.001 for all, Table 3). Table 4 shows that in Intervention Group mean T0 values for Fat Index (p = 0.016) were higher, due to more frequent consumption of pizza and kebab (p = 0.036) and meat pies and pastries (p < 0.001). Furthermore, although Sugar Index did not differ between the study groups, the consumption frequency of sweet pastries was lower in Intervention Group (p = 0.028). These baseline differences especially in fiber containing foods were taken into account in statistical analyses. In the analysis of covariance, adjustment of baseline values helps to account for coincidental baseline imbalances between study groups [38]. 

**Table 3:** **Consumption frequencies (days/week) of cereal index, fruit and vegetable index and food items included within**^**1**^

		**T0**		**T1**		**T2**	
		**mean**	**SD**	**mean**	**SD**	**mean**	**SD**
**Cereal Index**	Control	2.48	1.39	3.79*	1.39	2.98*†	1.34
	Intervention	3.02	1.46	3.94*	1.35	3.3*†	1.43
	Difference (p)	<0.001		0.199		0.006	
Rye and crisp bread	Control	3.51	2.39	4.62*	1.72	3.81†	1.82
	Intervention	4.47	2.25	4.59	1.86	4.00*†	1.96
	Difference (p)	<0.001		0.840		0.258	
Mixed bread	Control	2.54	2.05	2.65	1.64	2.69	1.51
	Intervention	3.15	2.21	2.78*	1.72	2.84*	1.67
	Difference (p)	0.001		0.361		0.253	
Porridges and cereals	Control	1.45	2.07	4.15*	2.15	2.47*†	2.08
	Intervention	1.54	2.00	4.46*	2.09	3.06*†	2.21
	Difference (p)	0.589		0.076		0.001	
**Fruit and Vegetable Index**	Control	2.24	1.68	2.79*	1.6	2.34†	1.53
	Intervention	2.95	1.82	2.66*	1.54	2.46*†	1.51
	Difference (p)	<0.001		302		0.354	
Fruit and berries	Control	1.96	1.81	2.31*	1.71	2.17	1.62
	Intervention	2.58	2.04	1.94*	1.48	2.03*	1.56
	Difference (p)	<0.001		0.006		0.308	
Fresh vegetables and salads	Control	2.53	2.03	3.29*	2.04	2.52†	1.82
	Intervention	3.34	2.12	3.38	2.16	2.91*†	1.95
	Difference (p)	<0.001		0.601		0.014	

^1^ At baseline (T0), 8 weeks of military service (T1) and 6 months of military service (T2) in the control and intervention groups (n = 604).

* Mean value significantly different from that at baseline (T0, p < 0.05).

† Mean value significantly different from that at 6 months (T1, p < 0.05).

**Table 4:** **Consumption frequencies (days/week) of fat index and sugar index and food items included within**^**1**^

		**T0**		**T1**		**T2**	
		**mean**	**SD**	**mean**	**SD**	**mean**	**SD**
**Fat Index**	Contol	0.86	0.67	0.68*	0.63	0.79†	0.79
	Intervention	1.00	0.72	0.55*	0.52	0.69*†	0.78
	Difference (p)	0.016		0.007		0.133	
French fries	Contol	1.06	1.20	0.61*	0.92	0.84*†	1.02
	Intervention	1.25	1.17	0.53*	0.89	0.71*†	1.05
	Difference (p)	0.064		0.315		0.123	
Potato chips	Contol	0.83	1.01	1.11*	1.16	0.85†	1.07
	Intervention	0.77	0.90	0.74	0.93	0.81	1.05
	Difference (p)	0.475		<0.001		0.716	
Pizza and kebab	Contol	1.09	1.11	0.81*	0.97	1.02†	1.14
	Intervention	1.29	1.17	0.74*	0.95	0.85*	1.04
	Difference (p)	0.036		0.338		0.052	
Hamburgers and hot dogs	Contol	0.95	1.06	0.55*	0.85	0.72*†	0.99
	Intervention	1.01	1.15	0.46*	0.73	0.59*†	0.99
	Difference (p)	0.520		0.183		0.158	
Meat pies and pastries	Contol	0.41	0.80	0.34	0.77	0.54†	0.91
	Intervention	0.74	1.03	0.29*	0.60	0.51*†	0.94
	Difference (p)	<0.001		0.387		0.729	
**Sugar Index**	Control	1.42	0.88	1.89*	0.95	1.61*†	0.84
	Intervention	1.37	0.83	1.75*	0.90	1.61*†	0.93
	Difference (p)	0.483		0.082		0.968	
Sugar-sweetened soft drinks	Control	2.61	1.96	3.05*	1.93	2.6†	1.74
	Intervention	2.80	2.07	2.73	1.86	2.54*†	1.92
	Difference (p)	0.274		0.048		0.678	
Sweet pastries	Control	1.66	1.66	1.97*	1.39	1.98*	1.38
	Intervention	1.37	1.5	1.65*	1.38	2.03*†	1.55
	Difference (p)	0.028		0.006		0.711	
Desserts	Control	0.64	1.08	2.02*	1.22	1.81*†	1.3
	Intervention	0.77	1.03	1.58*	1.20	1.74*†	1.33
	Difference (p)	0.139		<0.001		0.558	
Sweets	Control	1.69	1.54	2.61*	1.77	2.03*†	1.48
	Intervention	1.64	1.47	2.61*	1.79	2.11*†	1.59
	Difference (p)	0.716		0.982		0.536	
Chocolate	Control	1.17	1.44	1.81*	1.63	1.47*†	1.3
	Intervention	1.09	1.28	1.77*	1.62	1.4*†	1.47
	Difference (p)	0.495		0.793		0.572	

^1^ At baseline (T0), 8 weeks of military service (T1) and 6 months of military service (T2) in the control and intervention groups (n = 604).

* Mean value significantly different from that at baseline (T0, p < 0.05).

† Mean value significantly different from that at 8 weeks (T1, p < 0.05).

### Intervention effects on food consumption

**Figure 2:**
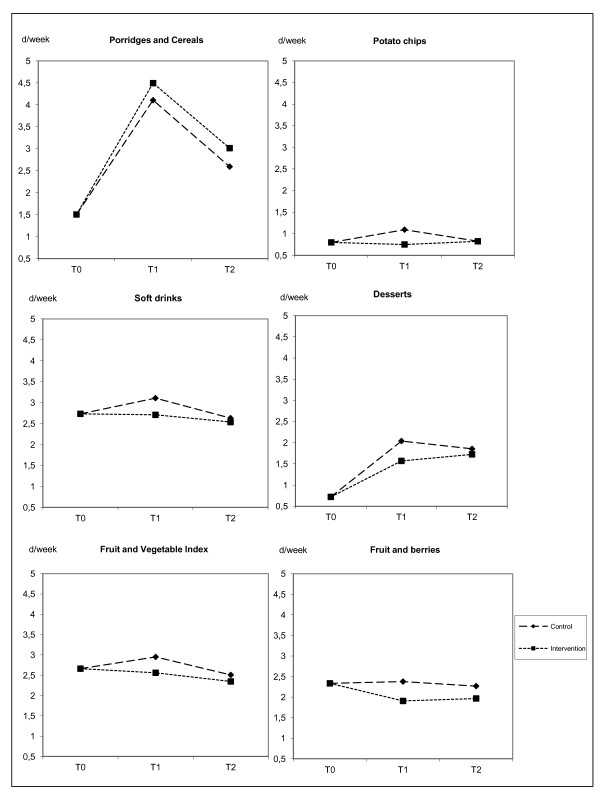
**Profile plots of outcome variables with significant intervention main effect and time-intervention interaction.** For porridges and cereals p = 0.006 for intervention effect and p = 0.044 for time-intervention interaction. For potato chips p = 0.01 for intervention effect and p < 0.001 for time-intervention interaction. For soft drinks p = 0.042 for intervention effect and p = 0.024 for time-intervention interaction. For desserts p = 0.001 for intervention effect and p < 0.001 for time-intervention interaction. For Fruit and Vegetable Index p = 0.005 for intervention effect and p = 0.005 for time-intervention interaction. For fruit and berries p < 0.001 for intervention effect and p = 0.002 for time-intervention interaction.

Food consumption patterns with a significant intervention main effect and time-intervention interaction are shown in Figure 2. These effects indicate different consumption patterns in the course of follow-up between the study groups. Differences positive for Intervention Group can be seen in individual food items. Porridges and cereals were consumed more frequently in Intervention Group during follow-up (p = 0.006 for intervention effect, p = 0.044 for time-intervention interaction). During the first follow-up period to eight weeks, consumption frequencies increased especially for potato chips (p = 0.01 for intervention effect, p < 0.001 for time-intervention effect) and soft drinks (p = 0.042 for intervention effect, p = 0.024 tor time-intervention interaction) and also for desserts (p = 0.001 for intervention effect, p < 0.001 for time-intervention interaction) in Control Group when compared to Intervention Group.

At the same time in Intervention Group, Fruit and Vegetable Index (p = 0.005 for intervention effect, p = 0.005 for time-intervention interaction) and consumption frequency of fruit and berries (p < 0.001 for intervention effect, p = 0.002 for time-intervention interaction) decreased from the first time point to 8 weeks whereas respectively they increased and remained stable in Control Group.

In addition, a main intervention effect was found for Fat Index (p = 0.016), indicating a lower consumption level of fatty foods in Intervention Group during follow-up. At six months follow-up, Cereal Index (p = 0.006) and the consumption frequency of fresh vegetables and salads (p = 0.014) was higher in Intervention Group than Control Group.

### The influence of military service on food consumption

The analysis of covariance revealed that food consumption changes clearly in the course of military service and compared to civilian life (Tables 3 &4). A highly significant main effect for follow-up time was found for Cereal Index, Fruit and Vegetable Index, Sugar Index and all the food items included in the indexes (p < 0.001 for all). Also, the effect was significant for Fat Index (p = 0.021), potato chips (p < 0.001), hamburgers and hot dogs (p = 0.026) and meat pies and pastries (p = 0.009).

## Discussion

In this real world non-randomized intervention to promote healthy eating in military setting, we found significant intervention results mostly in favor of Intervention Group. Measured by food indexes and individual food items, Intervention Group showed positive changes in several cereal fiber, fat and sugar containing foods compared to Control Group. Still, the intervention was not successful in increasing fruit and vegetable intake. These findings were not compromised by some significant differences between study groups at baseline, as these differences were taken into account in statistical analyses [38].

The positive results supported largely the nutritional goals of the intervention. Increasing fruit and vegetable consumption was not achieved contrary to a Danish military refectory intervention [28]. This result is unfortunate because earlier we have found that before service only 13% of young men ate vegetables daily and as few as 8% fruits and berries [12]. Earlier soldiers’ vegetable consumption has been shown to be low not only in Finland [11] but also in Norway [39,40]. One reason to possibly explain this result is the renovation of one of the garrison refectories during the study. This may have affected especially serving fresh salads and fruit because the refectory had to operate under field conditions. Also, fruit and vegetable consumption can be challenging to assess by self-reported measures especially among young men. Nevertheless, several intervention actions aimed to increase the supply and also consumption of fruit and vegetables.

A multitude of actions was developed in the intervention in the two main military eating environments. The actions were creative, diverse and can be divided into two categories: some were apparent and could be measured quantitatively. Others were less-visible to improve the quality of the food supply. The distinction has importance because the first-mentioned were the main focus of this study when using eating frequencies as main outcome measures. The last-mentioned can also improve the diet of conscripts but are not easily assessed by self-report measures. However, all actions aimed to promote healthy food supply and to meet the nutritional goals of the intervention. To examine the complete effect of the intervention, dietary habits are to be explored in more detail and potentially in relation to health risk factors [13].

Other dietary environmental interventions in the military setting can be considered with regards to these results. In the United States, new recipes for healthier food items were developed in order to decrease soldiers’ fat, cholesterol and sodium intake. This was successful as acceptability was positive for the new foods developed specifically for institutional eating at an army garrison refectory [41]. Sporul et al. [27] found that it is challenging to influence soldiers’ food choices with nutrition labeling of healthy foods at point-of-purchase. Healthy food labeling was reinforced with promotional posters but still sensory attributes such as taste, quality and appearance influenced meal selection most.

Environmental dietary interventions can be conducted also at the workplace or school setting. Intervening can involve physical and informational environments with the twofold aim of increasing the availability of healthy foods and providing education and support for health choices [42]. These settings lack the controlled environment where physical fitness is of importance. Also, military service is a period of institutional life where actions are externally directed. Individual’s possibilities of making independent decisions are limited. This applies also to eating because completely free dietary choices are unfeasible. The peer group is especially important in adolescence and it has major influence in developing both eating habits and lifestyles [43]. The role of the peer group may be even stronger in the military setting where individuality is diminished and actions directed. As dietary interventions can be more effective for young females [30], actions need to be targeted directly for males and gender specificity of actions needs to be improved especially in this setting. In all, improving implementation and ensuring maintenance of the positive achievements is important [43].

Other military interventions have focused on individual dietary education while tackling overweight and obesity. Approaches with dietary education and restriction of energy intake have resulted in reduction of body weight [19,22]. Also, behavioral internet treatment has been successful in weight loss, increasing fruit and vegetable consumption and decreasing snacking [44]. When dietary education was combined with a fitness program, positive results were achieved. The exercise-plus-diet intervention group lost weight and reduced energy intake, the proportion of fat and saturated fat in the diet. Also they increased fiber intake notably by increasing bread, fruit and vegetable consumption [24].

Our study confirms earlier findings [13] suggesting that military service per se is a significant intervention for conscripts also in terms of diet. Both study groups exhibited short and long-term changes including increased consumption of cereal foods and a decrease in fatty foods. However, the effect of service is not only a positive one as consumption of sugar-rich foods increases [13]. This could be difficult to influence because the military affects also food attitudes: craving for sweet foods, using food as a reward and as a source of pleasure increases [14]. However, the intervention was successful in decreasing consumption of several sugar-rich foods.

One issue to be considered is that first measurement time point (T0) was not identical in both groups as in Control Group the study questionnaire was sent home and responding took place before entering service. Thus, the time frame of responding expands from less than one month before military service to entering service. Respectively, Intervention Group responded at the beginning of military service. They were instructed to answer retrospectively regarding civilian life before military service. Advantageously, the retrospective time period is relatively short but this fact may affect and thus limit the interpretation of results. For collecting dietary data, a frequency method is applicable in short-term recall as it produces less excess in overestimation, within-person error and interpersonal variation [45] and it has also been used [46,47]. The issue was accounted in analyses when baseline consumption frequency was adjusted for as a covariate in the repeated measures ancova. This is an advisable procedure if there is an association with outcome measures [38], as was the case here (p < 0.01 for all, data not shown), especially when analyzing change from baseline and with continuous outcome measures [38].

## Conclusions

This study shows that an intervention promoting the supply of healthy foods in the military setting affects conscripts’ food choices. The intervention had positive effect on consumption frequency of porridges and cereals, fatty foods, and sugar-rich foods, such as soft drinks and desserts. These changes can be achieved by intervening in the main eating environments and food providers in the military. However, fruit and vegetable consumption is more difficult to influence through this environment. Further studies should also incorporate components targeting specifically promotion of individual motivation to choose these food items. With time, these young men become adults. Intervening on conscripts’ eating habits with a positive effect can have significant public health implications and relevance later in adulthood. Finding effective ways to influence and reinforce sustainably their healthy eating habits remains a key issue.

## Competing interests

The authors declare that they have no competing interests.

## Authors’ contributions

CMLB: Designed the study, collected data, conducted analyses and wrote the manuscript. ML-K: Designed the study and analyses and critically reviewed the manuscript. PP: Acted as the statistical expert of the study, conducted analyses and critically reviewed the manuscript. MK: Designed the study, collected data and critically reviewed the manuscript. PJ: Designed the study and analyses and critically reviewed the manuscript. PA: Designed the intervention and analyses and critically reviewed the manuscript. All authors read and approved the final manuscript.
